# Practicalities of mapping PM_10_ and PM_2.5_ concentrations on city-wide scales using a portable particulate monitor

**DOI:** 10.1007/s11869-016-0394-3

**Published:** 2016-02-21

**Authors:** Michael E. Deary, Samantha J. Bainbridge, Amy Kerr, Adam McAllister, Thomas Shrimpton

**Affiliations:** Department of Geography, Faculty of Engineering and Environment, Northumbria University, Ellison Building, Newcastle upon Tyne, NE1 8ST UK

**Keywords:** PM_10_, PM_2.5_, DustMate, Osiris, Ambient monitoring

## Abstract

Fine particulate matter is considered to be the most significant ambient air pollutant in terms of potential health impacts. Therefore, it is important that regulators are able to accurately assess the exposure of populations to PM_10_ and PM_2.5_ across municipal areas. We report on the practicalities of using a laser light scattering portable particulate monitor (Turnkey Instruments DustMate), in combination with a GPS, to map PM_10_ and PM_2.5_ concentrations on city-wide scales in Newcastle upon Tyne/Gateshead (UK), during a series of walking surveys. A heated inlet is necessary to remove moisture droplets from the sampled air prior to analysis by the instrument, though this also results in the loss of volatile particulate components, particularly from the PM_2.5_ fraction. A co-location calibration study was carried out with a reference urban background Tapered Element Oscillating Micro-Balance/Filter Dynamics Measuring System (TEOM-FDMS) system in Newcastle that is part of the UK’s Automatic Urban and Rural Network (AURN) of air quality monitoring stations. For PM_10_, orthogonal regression of the DustMate against TEOM-FDMS data gave a slope and intercept of 1.02 ± 0.06 and −3.7 ± 1.2, respectively (*R* ^2^ = 0.73), whereas for PM_2.5_, the respective values were 0.78 ± 0.06 and −0.63 ± 0.55 (*R* ^2^ = 0.79). These parameters are comparable to literature calibration studies using this technology. There was good agreement between simultaneous samples taken using two DustMate instruments: for PM_10_, a slope and intercept of 1.05 ± 0.03 and 0.36 ± 0.5, respectively (*R* ^2^ = 0.73), were obtained, whereas for the PM_2.5_, the respective values were 0.79 ± 0.01 and 0.19 ± 0.06 (*R* ^2^ = 0.86). Correction factors based on the slope and intercepts obtained from the calibration exercise were applied to raw data collected from the DustMate. An annually-normalised correction procedure was then used to account for different background particulate concentrations on different sampling days. These corrected PM_10_ and PM_2.5_ concentrations and corresponding GPS coordinates were displayed on a base map using Google Fusion Tables and Google Earth Professional. Almost all areas surveyed in Newcastle/Gateshead were well below the EU Air Quality Standards for PM_10_ and PM_2.5_.

**Electronic supplementary material:**

The online version of this article (doi:10.1007/s11869-016-0394-3) contains supplementary material, which is available to authorized users.

## Introduction

The increasing portability of analytical instruments capable of monitoring airborne pollutants has made the concept of dynamic mapping of air pollution in towns and cities a viable proposition, possibly even in real time (Moltchanov et al. 2015). This has already been realised for pollutants that can be measured using electrochemical techniques, where improved sensitivity, combined with a high level of portability, has allowed such sensors to be used alongside GPS devices to map pollution concentrations in cities including Cambridge, Valencia and Lagos (Mead et al. 2013). Typically, the pollutants that can be measured with such devices are ozone, nitrogen dioxide, nitric oxide and carbon monoxide (Mead et al. 2013). There are still cross-sensitivity issues with these devices, particularly between nitrogen dioxide and ozone, though this can be compensated for by measuring both pollutants simultaneously and then applying a correction algorithm (Lin et al. 2015). Some promising work has been carried out in this field that raises the prospect of these cheap and unobtrusive sensors being utilised in an array of fixed and mobile locations that, combined with smartphone technology, may allow detailed real-time pollution concentrations to be monitored at high resolution on city-wide scales (Moltchanov et al. 2015; Kumar et al. 2015). Detailed mapping of airborne pollutant concentrations in real time or otherwise will allow greater refinement of pollutant exposure estimation for population groups, as well as facilitating the identification of areas of poor air quality (Moltchanov et al. 2015). Such technology also has application in determining the personal exposure of people throughout the day as they encounter a range of pollution microenvironments at home, at work and in recreational/leisure facilities, thus providing a refined estimation of potential health impacts that is not simply reliant on a generalised exposure derived from one or two fixed ambient air quality stations and associated dispersion modelling (de Nazelle et al. 2013; Gulliver and Briggs 2004; Gerharz et al. 2009; Buonanno et al. 2011, 2014; Deary and Uapipatanakul 2014).

Whilst this is encouraging from the perspective of more effectively characterising the exposure of populations to nitrogen oxides, ozone and carbon monoxide, which are major pollutants in many cities globally, the monitoring of particulate pollution in a similar way is not so straightforward. Considerable progress has been made in designing real-time portable particulate monitors, usually based on light scattering technology; however, sensitivity requirements mean that they remain relatively bulky and quite costly in comparison to the electrochemical sensors. There are also significant technical issues in their use, specifically related to calibration and equivalence with other particulate monitoring techniques. One major issue is the necessity to use a heated inlet to vaporise fine water droplets that would otherwise contribute to the particulate counts but which also serves to remove a significant proportion of the volatile organic component of the sample. It is important that such problems are overcome so that the mapping capability for particulates matches those of other pollutants because PM_10_ and, especially, PM_2.5_ are the ambient air pollutants considered to represent the most significant risk to health (Anderson et al. 2013; Kelly and Fussell 2015; Sapkota et al. 2012; Fann and Risley 2013).

In this paper, we present data from a study using a Turnkey DustMate portable particulate monitor in combination with a hand-held GPS to map PM_10_ and PM_2.5_ concentrations in Newcastle upon Tyne/Gateshead, UK. We report on the calibration against a reference method (Tapered Element Oscillating Micro-Balance/Filter Dynamics Measuring System (TEOM-FDMS)) and the results of between-sampler comparisons. Illustrative maps of ambient particulate concentrations, plotted using Google Fusion Maps and Google Earth Pro, are presented to demonstrate the application of our approach.

## Methodology

### Overview of the Turnkey Instruments DustMate particulate monitor

The DustMate is a lightweight portable version of the Osiris laser light scattering particulate monitor that is capable of measuring total suspended particulates (TSP), PM_10_, PM_2.5_ and PM_1_ with a resolution of 0.1 μg m^−3^. Air is drawn into the instrument at a rate of 0.6 L min^−1^, and the flow is configured so that only one particle is illuminated by the laser light beam (670 nm) at any particular moment in time. The signal obtained from the diffraction of these individual particles is then converted to an equivalent mass using a look-up table. The instrument only measures diffraction angles between 0 and 10°, over which range diffraction is independent of particulate composition (Turnkey Instruments 2002).

This technology, in the form of the Osiris monitor, has been widely used for ambient particulate monitoring in urban environments (King and Dorling 1997) as well as in major fire incidents (Griffiths et al. 2015). It has also been used for investigating individual exposure to particulates during different modes of travel (Gulliver and Briggs 2004, 2007). The DustMate itself, whilst intended primarily for workplace situations, has nevertheless been used to monitor ambient concentrations (Liu et al. 2004; Chen et al. 2015; Mustapha et al. 2011; Kim et al. 2008; Duché and Beltrando 2012), as well as to assess individual exposure in different pollution microenvironments (Gulliver and Briggs 2007; Li et al. 2006). There have also been more exotic applications: for example, being attached to a Cessna light aircraft to measure particulate concentrations in north-west Germany during and after the Eyafjallajökull volcanic eruption in 2010 (Weber et al. 2012). The DustMate has an optional battery-powered heated inlet comprising two cylindrical ceramic heating elements attached to an axially located steel inlet tube. The incoming air is heated to approximately 50 °C which vaporises moisture droplets that would otherwise have contributed to the particulate concentration.

### Instrument performance and calibration

#### Effect of heated inlet use

PM_10_, PM_2.5_ and PM_1_ concentrations were measured over a range of meteorological conditions with and without the heated inlet in operation: three alternating 15-min sampling periods were used for each, with an averaging time of 1 min.

#### Between-sampler comparability

Reproducibility of PM_10_ and PM_2.5_ measurements was determined by deploying two DustMate monitors to take simultaneous measurements during nine separate monitoring exercises, totalling 470 individual minute averages. The monitoring was carried out in Sunderland (UK) city centre and comprised the same walk carried out during morning (7.30 to 8.30), mid-day (11.30 to 12.30) and late afternoon (16.30 to 17.30), repeated on the Friday of three consecutive weeks.

#### Instrument performance compared to the TEOM-FDMS reference method

A co-location calibration study was carried out for the DustMate and an urban background TEOM-FDMS analyser operated by Newcastle City Council as part of the UK Automatic Urban and Rural Network (AURN) of air quality monitoring stations (DEFRA 2015). Data was collected for 41-h-long periods at various times throughout the day (between 9.00 and 20.00) and during a range of different meteorological conditions. Calibration factors (slope and intercept) were determined from orthogonal (major axis) regression of DustMate against TEOM-FDMS data for both PM_10_ and PM_2.5_ (EC Working Group on Guidance for the Demonstration of Equivalence 2010).

### Application of the DustMate to map PM_10_ and PM_2.5_ concentrations on a city-wide scale

Walking surveys were carried out in Newcastle/Gateshead on weekdays between the period 09 Jun 2015 to 29 Jun 2015 inclusive, each between 7.30 and 9.30 a.m. (local time) to coincide with the morning rush hour. Each walk took place in a different area of the city.

DustMate monitors are designed for hand-held operation, but this is not practical for the longer-term monitoring exercises carried out in this study, and so a specifically designed polyurethane foam lined box was used to house the instrument and its battery and also the battery used to power the heated inlet. The controls were accessible via a slot cut in the box, and the inlet was attached through a hole at the top of the box (see Figs. S1, S2, S3 and S4 in the Supplementary Material). The box was carried in a 30-L capacity backpack. A Trimble Juno SB GPS, installed with TerraSync Centimeter Edition, was used to record the latitude/longitude (decimal degrees) positions of the monitors every 5 s (the ‘feature logging’ setting was set as ‘time’). The DustMate internal clock was synchronised to that of the GPS.

Particulate concentrations were matched to location by creating a spreadsheet of particulate concentrations and corresponding measurement time and then defining this as a database in Microsoft Excel. The VLOOKUP function was used to match individual GPS coordinates to particulate concentrations using time as the common parameter. The time outputs for both devices had to be re-coded to a common format (see sample spreadsheet in the Supplementary Material).

In order to plot the data on Google Maps/Google Earth, it is preferable to use line segments rather than individual points: these can be created from the GPS coordinates of two adjacent points, by converting to KML line code using the formula shown in the sample spreadsheet included in the Supplementary Material. The data was uploaded to Google Fusion Tables (Google 2015) as a csv file. Line segments are overlaid onto a base map and colour coded according to particulate concentration. A complete KML file, or a KML link, can also be exported from Google Fusion Tables for import into Google Earth Professional which allows higher-resolution images to be output (maximum of 4800 × 3195 pixels), as used in this paper.

## Results and discussion

### Effect of using the heated inlet

Table 1 shows the effect of heated inlet use on the measured concentrations of PM_10_, PM_2.5_ and PM_1_ under a range of meteorological conditions. It is clear that in high humidity and poor visibility conditions, there is a significant discrepancy between the concentrations measured for heated and non-heated inlet use. The first two entries in the table, corresponding to poor to moderate visibility conditions and high relative humidity, show that concentrations for heated conditions range between 13 and 23 % of those measured without the heated inlet for PM_10_, with a corresponding range of between 12 and 51 % for PM_2.5_. Without the heated inlet, it is likely that water droplets are contributing a significant proportion of the overall particle count. The situation is very different for conditions where there is better visibility and lower relative humidity (entries 3 to 6) where the concentration of PM_2.5_ measured with heated inlet ranges between 69 and 81 % of the value obtained without the heated inlet. The corresponding range for PM_10_ is 27 to 52 %, suggesting that the presence of airborne moisture droplets, even in low humidity/good visibility conditions, disproportionately affects the PM_10_ fraction. These results demonstrate the importance of using a heated inlet for the measurements so that the effect of meteorological conditions is negated and that users are not restricted to conditions of high visibility, as has been the case in some previous DustMate studies (Duché and Beltrando 2012). Nevertheless, use of the heated inlet is problematic because, in addition to the removal of moisture droplets, there is likely to be some loss of volatile components associated with the particulates.

**Table 1:** Comparison of particulate matter concentrations measured by the DustMate monitor with and without using the heated inlet under a variety of meteorological conditions

Sample details	Temp. (°C)	RH (%)	Precip.	WS (m s^−1^)	Vis. (km)	PM_10_( μg m^−3^)		PM_2.5_ (μg m^−3^)		PM_1_ (μg m^−3^)	
						NH	H	NH	H	NH	H
Northumbria University, 13 Feb 2015, mid-morning	4	93	No	3.6	4	159 (16.0)	21.3 (3.0)	106 (9.9)	13.2 (2.7)	36.1 (2.6)	5.5 (1.4)
Dunston Road, Gateshead; 14 Feb 2015, evening	6	93	No	2.1	1.8	201 (90)	46.1 (4.1)	37.9 (5.5)	19.3 (1.6)	11.1 (2.3)	3.5 (0.5)
New Bridge Street, Newcastle; 16 Feb 2015, evening.	4	87	No	4.4	10	17.7 (5.7)	7.0 (1.3)	4.3 (1.6)	3.1 (0.5)	0.9 (0.5)	0.6 (0.1)
Shieldfield, Newcastle, 20 Feb 2015, mid-morning	5	76	No	7.7	10	44.2 (3.6)	12.1 (1.6)	7.5 (0.3)	5.2 (0.3)	0.9 (0.1)	0.9 (0.1)
Jesmond By-pass, 10 Mar 2015, mid-day.	8	57	No	6.2	10	54.6 (6.1)	28.6 (4.6)	11.2 (0.5)	9.1 (0.6)	1.5 (0.2)	1.5 (0.1)
St Mary’s Place, Newcastle, 10 Mar 2015, mid-day.	8	57	No	6.2	10	42.1 (5.9)	18.1 (2.0)	9.4 (1.0)	6.9 (0.4)	1.4 (0.3)	1.2 (0.1)

Standard deviations are given in parentheses

*NH* non-heated inlet, *H* heated inlet, *RH* relative humidity, *WS* wind speed

### Between-sampler comparability

**Fig. 1:**
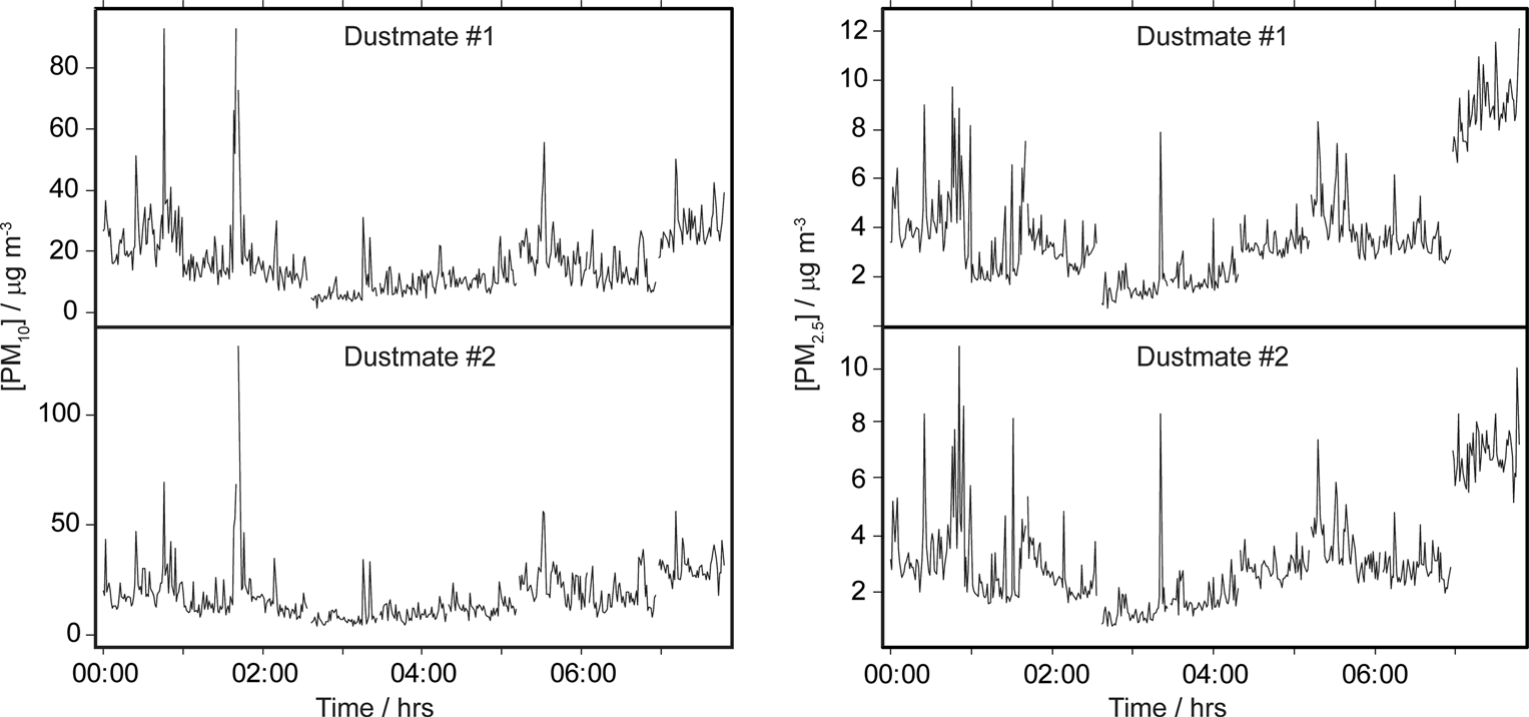
Comparison of PM_10_ and PM_2.5_ concentrations measured simultaneously by two DustMate instruments with heated inlets during a walking survey in Sunderland, UK. The time axis shows the cumulative hours/minutes of the survey (7 h and 47 min of data in tota, collected over 3 days). Each point is calculated from concentrations averaged over a 1-min sampling time

**Fig. 2:**
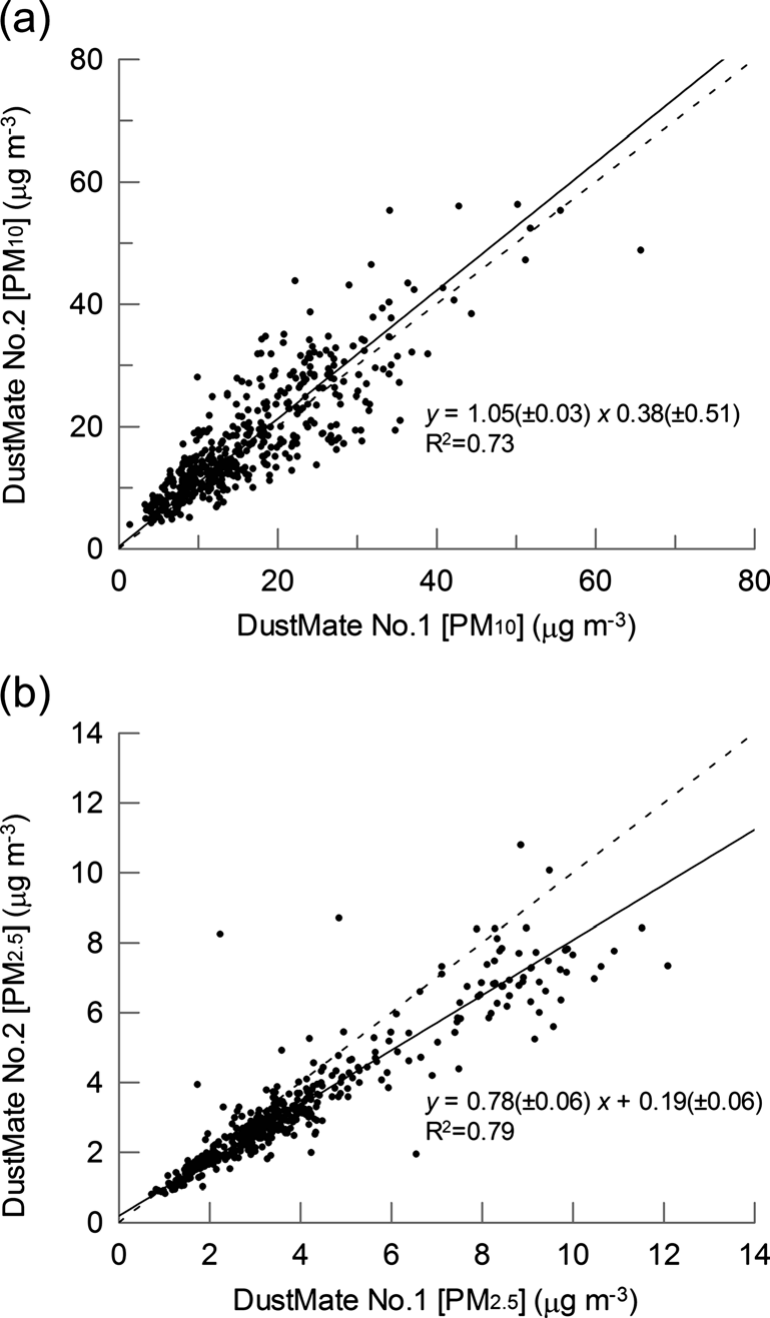
Scatterplot of **a** PM_10_ and **b** PM_2.5_ concentrations measured simultaneously by two DustMate instruments with heated inlets during a walking survey in Sunderland, UK. Each point is calculated from concentrations averaged over a 1-min sampling time. The *solid* *lines* are the best fit to the points using orthogonal regression and the equations shown on the plots. The *dashed lines* represent *y* = *x*

Figure 1 shows a comparison of the PM_10_ and PM_2.5_ concentration time series (1-min averages) recorded by two separate particulate monitors, each for nine separate walks that occurred on three separate days (three walks per day); the corresponding scatterplots are shown in Fig. 2. For PM_10_, orthogonal regression of the scatterplot gives a slope and intercept of 1.05 ± 0.03 and 0.36 ± 0.5, respectively (*R* ^2^ = 0.73), whereas for the PM_2.5_, the respective values were 0.79 ± 0.01 and 0.19 ± 0.06 (*R* ^2^ = 0.86). For PM_2.5_, the slope is less than unity; however, the most responsive monitor (no. 1) was used for all other monitoring reported in this paper. The *R* ^2^ value for the PM_10_ correlation was poorer than that for PM_2.5_; this has also been observed in a previous study (Halliburton et al. 2007). Overall, the results give reassurance that at a sampling time of 1 min, both monitors are registering the same variations in the ambient concentrations.

### Instrument performance compared to the TEOM-FDMS reference method

**Fig. 3:**
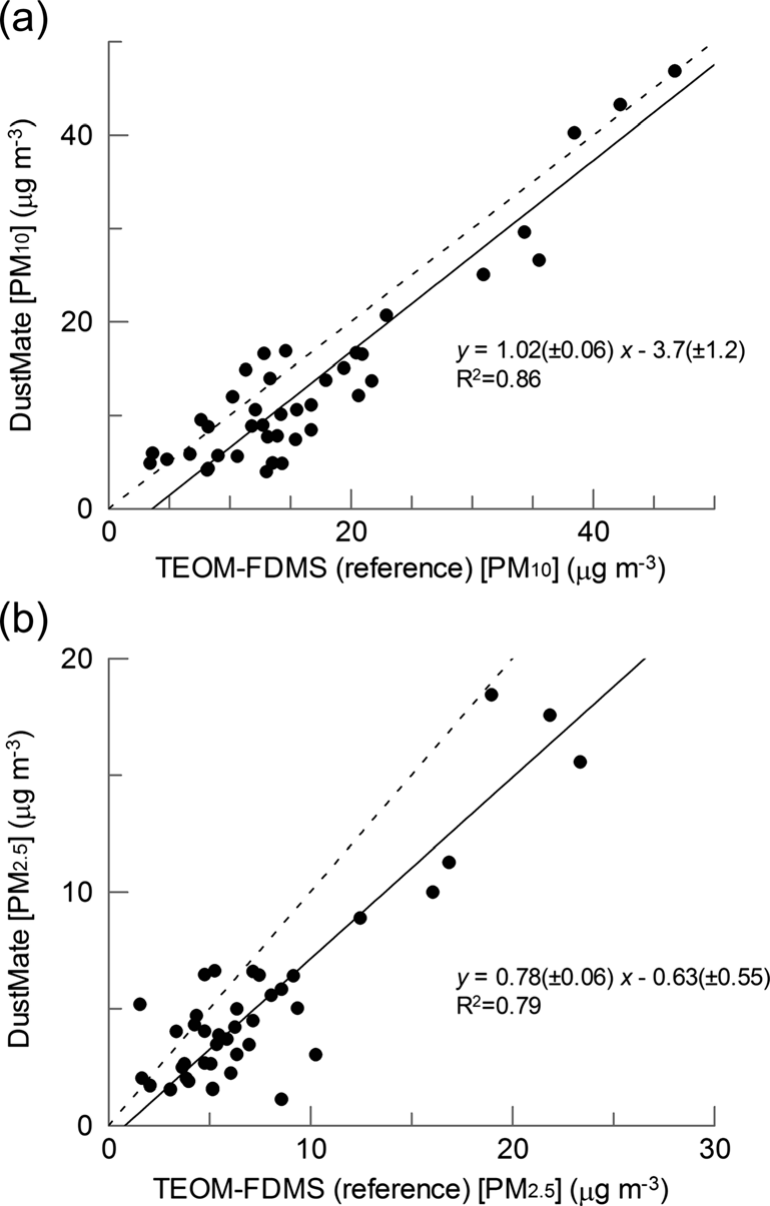
Calibration plots for **a** PM_10_ and **b** PM_2.5_ concentrations measured using a DustMate (with heated inlet) compared to the reference TEOM-FDMS method. Each point is calculated from concentrations averaged over a 1-h sampling time. The *solid lines* are the best fit to the points using orthogonal regression and the equations shown on the plots. The *dashed lines* represent *y* = *x*

Calibration plots for DustMate-measured against the TEOM-FDMS-measured PM_10_ and PM_2.5_ concentrations are shown in Fig. 3. Over the duration of the calibration study, the TEOM-FDMS-measured concentrations ranged between 3.5 and 46.8 μg m^−3^ for PM_10_ and 1.7 and 23.4 μg m^−3^ for PM_2.5_. For PM_10_, orthogonal regression gave a slope and intercept of 1.02 ± 0.06 and −3.7 ± 1.2, respectively (*R* ^2^ = 0.73), whereas for the PM_2.5_, the respective values were 0.78 ± 0.06 and −0.63 ± 0.55 (*R* ^2^ = 0.79). In literature DustMate/Osiris calibration studies for PM_10_, all conducted using a TEOM, which generally underestimates particulate concentrations compared to TEOM-FDMS (Favez et al. 2007), slopes of 1.03 (Gulliver and Briggs 2004), 0.70 ± 0.03 (Waldén et al. 2010) and 0.97 (Halliburton et al. 2007) were obtained. The latter two studies reported similar *R* ^2^ values to ours. As with our study, a negative intercept (−0.91) was observed in the Waldén study for PM_10_ (Waldén et al. 2010). For our PM_2.5_ calibration, the slope of 0.78 ± 0.06 compares to that of 0.56 ± 0.03 obtained by Walden (Waldén et al. 2010) and 0.68 from the study of Haliburton (though for a 24 h rather than 1 h mean) (Halliburton et al. 2007). These lower slopes for PM_2.5_ compared to PM_10_ reflect the higher proportion of volatile organics associated with the finer particulate fractions compared to PM_10_. PM_1_ is not measured by the TEOM-FDMS, and so, a direct comparison is not possible, but it is likely that the DustMate will underestimate the total PM_1_ by a similar or greater extent to PM_2.5_. The diurnally and seasonally varying volatile content of ambient particulate matter (Favez et al. 2007) is likely to influence the accuracy of the DustMate measurements when made with a heated inlet: significant loss of volatiles is likely during the heating process, particularly for PM_2.5_, when compared to TEOM-FDMS measurements. Future work will investigate alternative methods for removal of water droplets from sampled air, for example, Nafion/desiccant drying systems that might overcome problems encountered with loss of volatiles when using heated inlets (Grimm and Eatough 2009).

The variation in calibration data between different studies and also between different environments such as rural and urban (Halliburton et al. 2007) makes it desirable that in advance of ambient studies, such as those described in this paper, a field calibration is carried out in the specific study location over the same time period. Correction factors based on the slope and intercepts obtained from our calibration were applied to raw data collected from the DustMate.

### Sensitivity to pollution sources

**Fig. 4:**
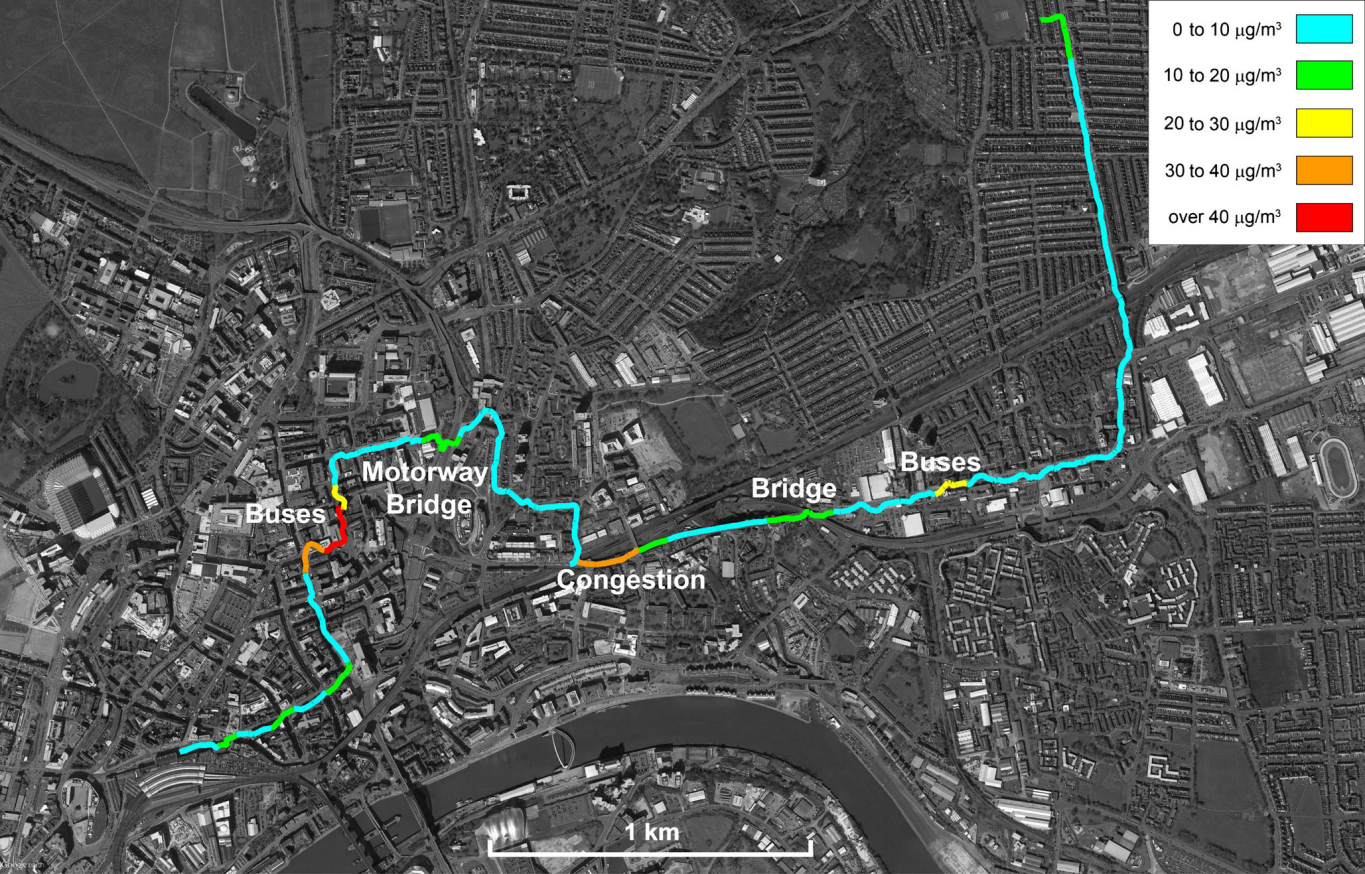
PM_10_ concentrations across a transect from the eastern suburbs to the centre of Newcastle upon Tyne UK, collected using a DustMate monitor with heated inlet on 17 Jun 2015. No normalisation procedure was applied to this data. Map produced using Google Fusion Tables and Google Earth Pro. Map data: Google, Landsat

Figure 4 shows a single walk in Newcastle from east to west, highlighting the coincidence of elevated PM_10_ concentrations with specific traffic features. Higher concentrations are encountered at a set of bus stops, at a bridge where traffic lanes have resulted in reduced road capacity, at the location of stationary traffic on the approaches to the city centre, at a pedestrian bridge over a motorway and in the city centre at a location with high bus throughput.

### Air quality mapping

One issue to be resolved before mapping the particulate concentrations was how individual walks on different days could be used together to create an overall map, given that prevailing meteorological conditions will affect background concentrations. A normalisation procedure was used, based on the approach used by local authorities in the UK to seasonally adjust NO_2_ diffusion tube data when this is available for less than a full year (DEFRA 2009). We used Eq. 1 to carry out the normalisation, where [FDMS_07.00–10.00Day_] is the average PM concentration measured by the AURN TEOM-FDMS in Newcastle over the specific 07.00 to 10.00 morning period during which an individual walk took place, and [FDMS_07.00–10.00Year_] is the annual average for all weekday 07.00 to 10.00 periods. The quotient in Eq. 1 ranged between 0.44 and 1.98, with a mean value of 0.78.

1 $$ {\mathrm{PM}}_{\mathrm{normalised}}=\frac{\left[{\mathrm{TEOM}\hbox{-} \mathrm{FDMS}}_{07.00-10.00\mathrm{Da}y}\right]}{\left[{\mathrm{TEOM}\hbox{-} \mathrm{FDMS}}_{07.00-10.00\mathrm{Year}}\right]}\cdot {\mathrm{PM}}_{\mathrm{non}\hbox{-} \mathrm{normalised}} $$

**Fig. 5:**
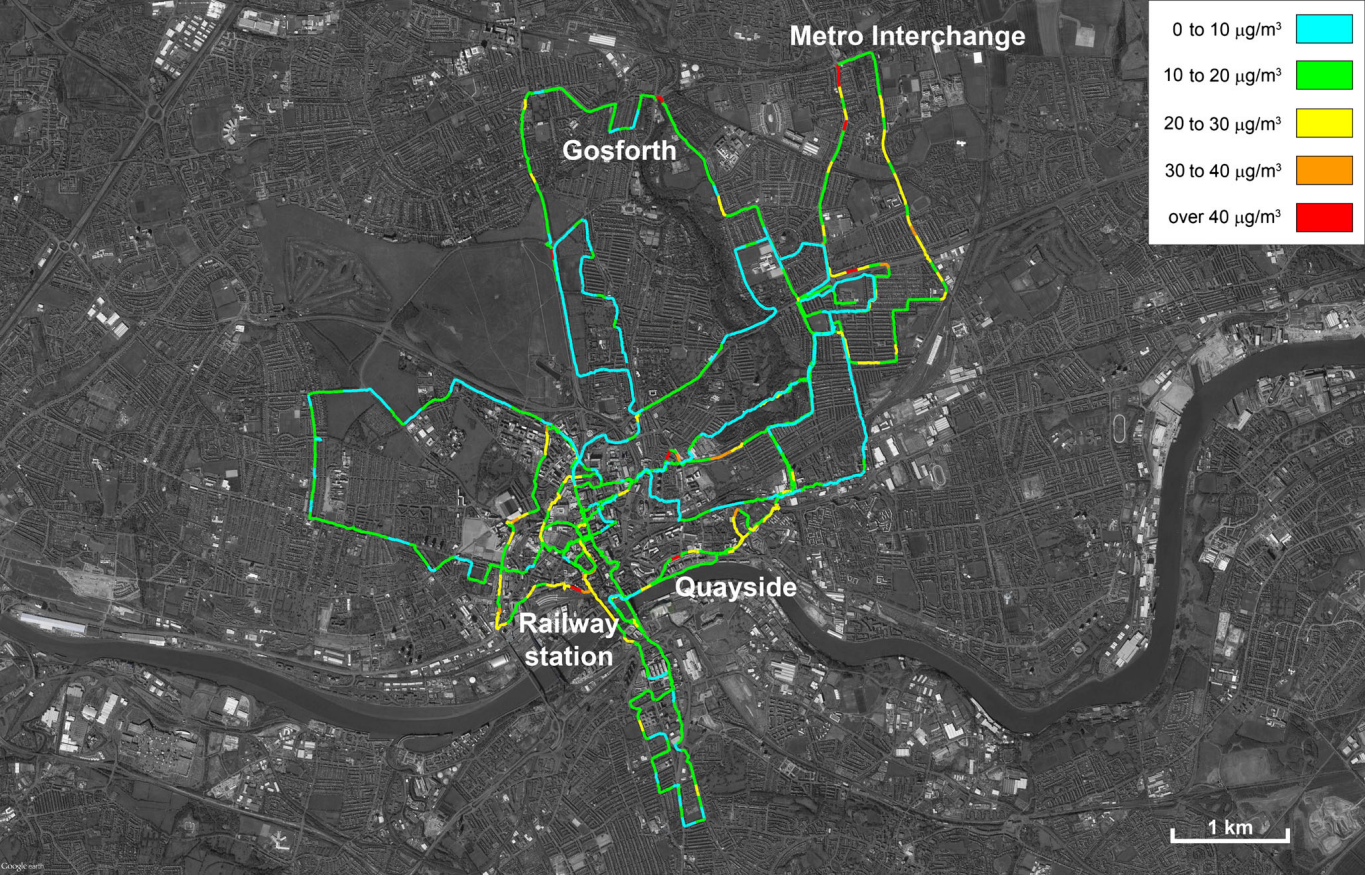
Annually normalised PM_10_ concentrations across Newcastle upon Tyne and Gateshead, UK, collected using a DustMate monitor with heated inlet during the period 09 Jun 2015 to 26 Jun 2015. Map produced using Google Fusion Tables and Google Earth Pro. Map data: Google, Landsat

The normalised data for PM_10_ has been plotted onto a base map for Newcastle/Gateshead in Fig. 5; the key indicates in red those areas that exceeded the European Union annual Air Quality Standard of 40 μg m^−3^ (The European Parliament and the Council of the European Union 2008). There are a small number of exceedances of the annual standard, notably the area adjacent to the railway station, the quayside area, the Metro (light rail system) interchange where significant roadworks were taking place and parts of the northern suburbs. More surveys in these areas would be necessary to confirm the existence of consistently elevated concentrations, with longer-term monitoring using a reference method required for regulatory purposes. Nevertheless, the identification of areas of elevated particulate concentrations, that might not have been indicated using modelling approaches alone, is a useful additional tool for those involved in local air quality management.

For PM_2.5_ concentrations, shown in Fig. S7 (Supplementary Material), there were no areas that exceeded the annual Air Quality Standard of 25 μg m^−3^. Raw data for PM_10_ and PM_2.5_ is mapped in Figs. S6 and S8 in the Supplementary Material, respectively, which also contains a link to the normalised data in Google Fusion Maps.

## Conclusions

This study has demonstrated how ambient PM_10_ and PM_2.5_ concentrations can be mapped on city-wide scales using portable particulate monitors in combination with a GPS. The approach presented in this paper has the potential to identify areas of elevated PM concentrations that might not have been detected when employing modelling approaches alone.

We have shown that in order for these monitors to be used over a range of meteorological conditions, prior removal of moisture droplets is necessary, in this case using a heated inlet. Nevertheless, the diurnally and seasonally varying volatile content of ambient particulate matter is likely to influence the accuracy of the DustMate measurements when made with a heated inlet; significant loss of volatiles is likely during the heating process, particularly for PM_2.5_, when compared to TEOM-FDMS measurements. There is scope for further work on alternative methods for removal of water droplets.

Finally, the relatively low cost of these portable particulate monitors (ca. $5k with heated inlet) presents municipal authorities with an opportunity to carry out high-resolution mapping of ambient particulate concentrations by enlisting walking commuters in different city areas. This could also form the basis of community or educational projects, especially since the results can be made publicly available using Google Maps, as we have demonstrated. Such initiatives have the potential to raise public awareness of air quality issues and to facilitate the involvement of local communities in schemes designed to reduce the levels of ambient air pollutants.

## Electronic supplementary material

Below is the link to the electronic supplementary material.

ESM 1 (DOC 39435 kb)

ESM 2 (XLSX 120 kb)
