# Supplementary material for: Practicalities of mapping PM10 and PM2.5 concentrations on city-wide scales using a portable particulate monitor
Source: Air Qual Atmos Health. 2016 Feb 21;9(8):923–30. doi: 10.1007/s11869-016-0394-3 (PMC5093208; doi:10.1007/s11869-016-0394-3)
Supplement: Supplementary file 1 — (DOC 39435 kb) [file 11869_2016_394_MOESM1_ESM.doc]

Supplementary material for Manuscript AIRQ-D-15-00177: Practicalities of mapping of PM10 and PM2.5 concentrations on city-wide scales using a portable particulate monitor

Link to Google Fusion Map for PM_2.5_

<https://www.google.com/fusiontables/DataSource?docid=1FyFO0Who9WGtkJyERftN6zV3nXaatJiDp9YBkYSQ>


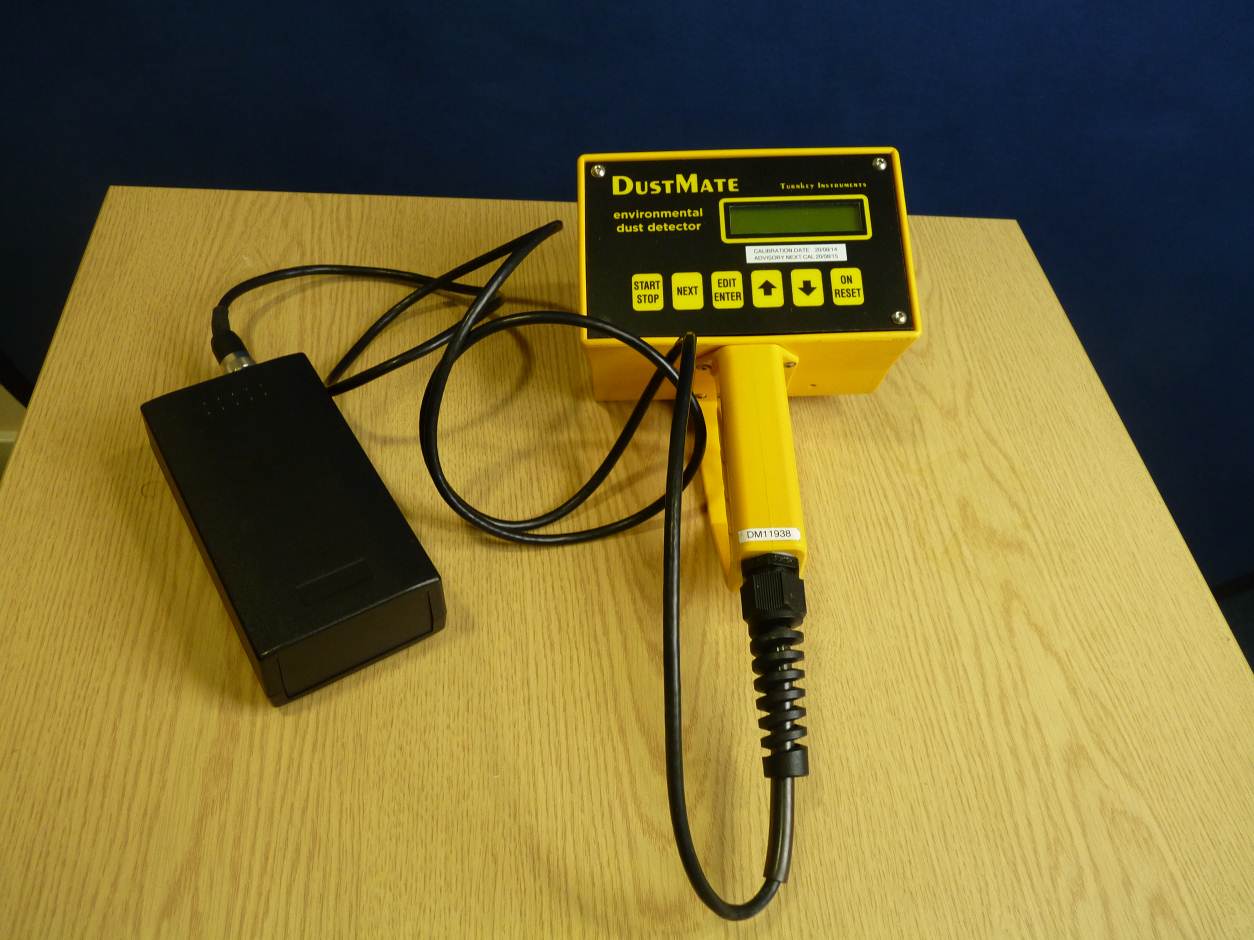


Figure S1: Turnkey Instruments DustMate environmental monitor and battery pack without heated inlet.


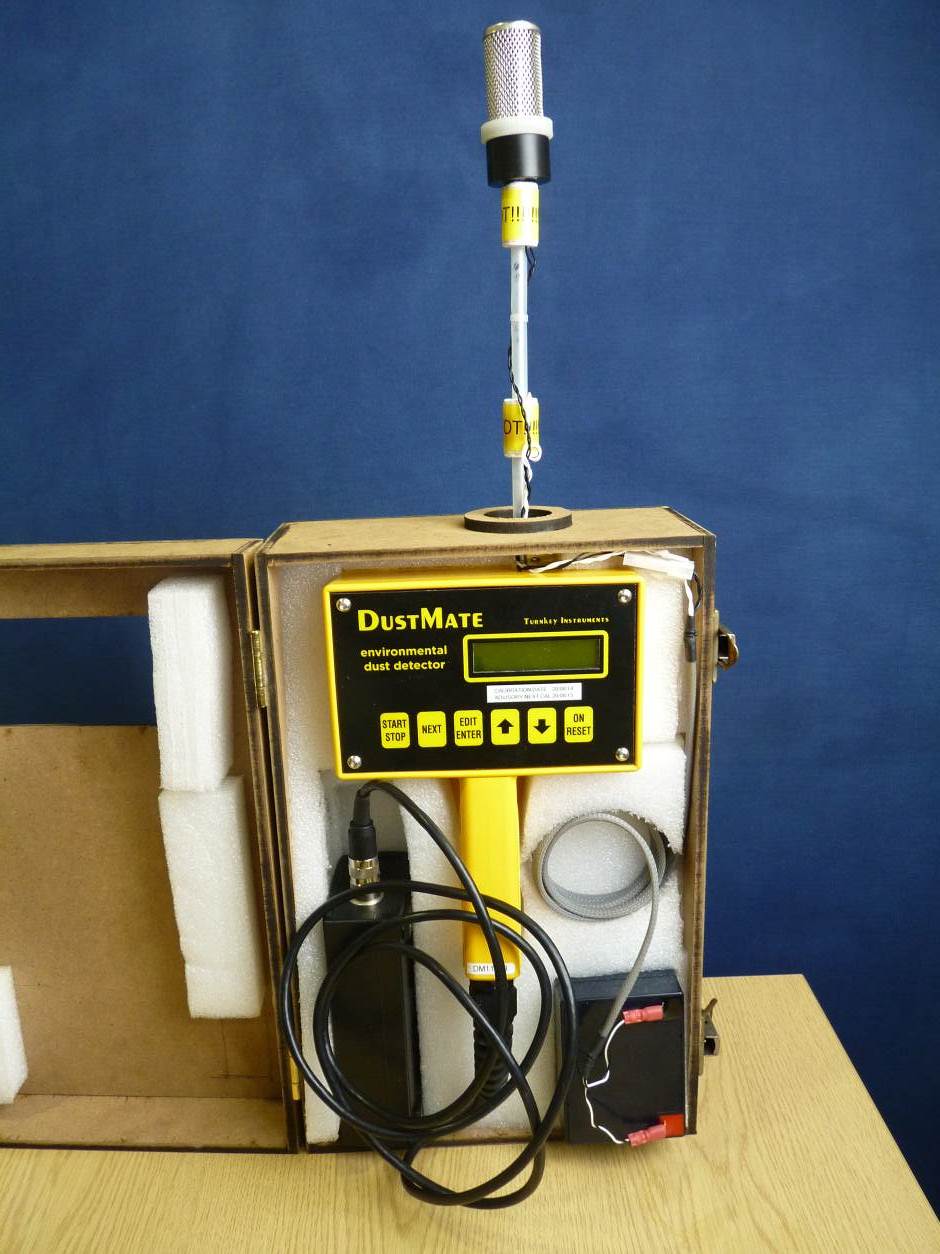


Figure S2: Polyurethane foam housing for DustMate monitor, battery, heated inlet and its battery.


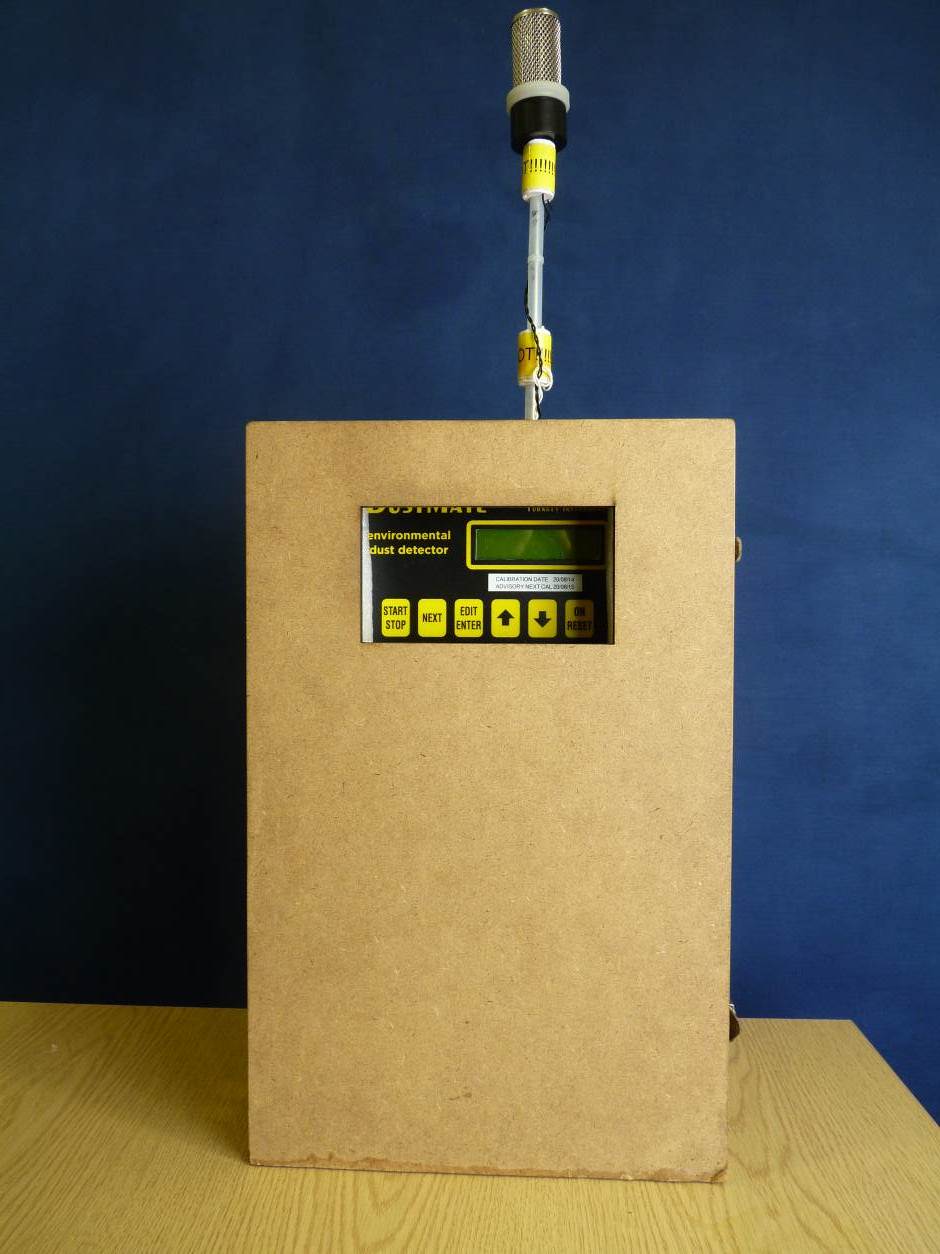


Figure S3: DustMate housing ready for use.


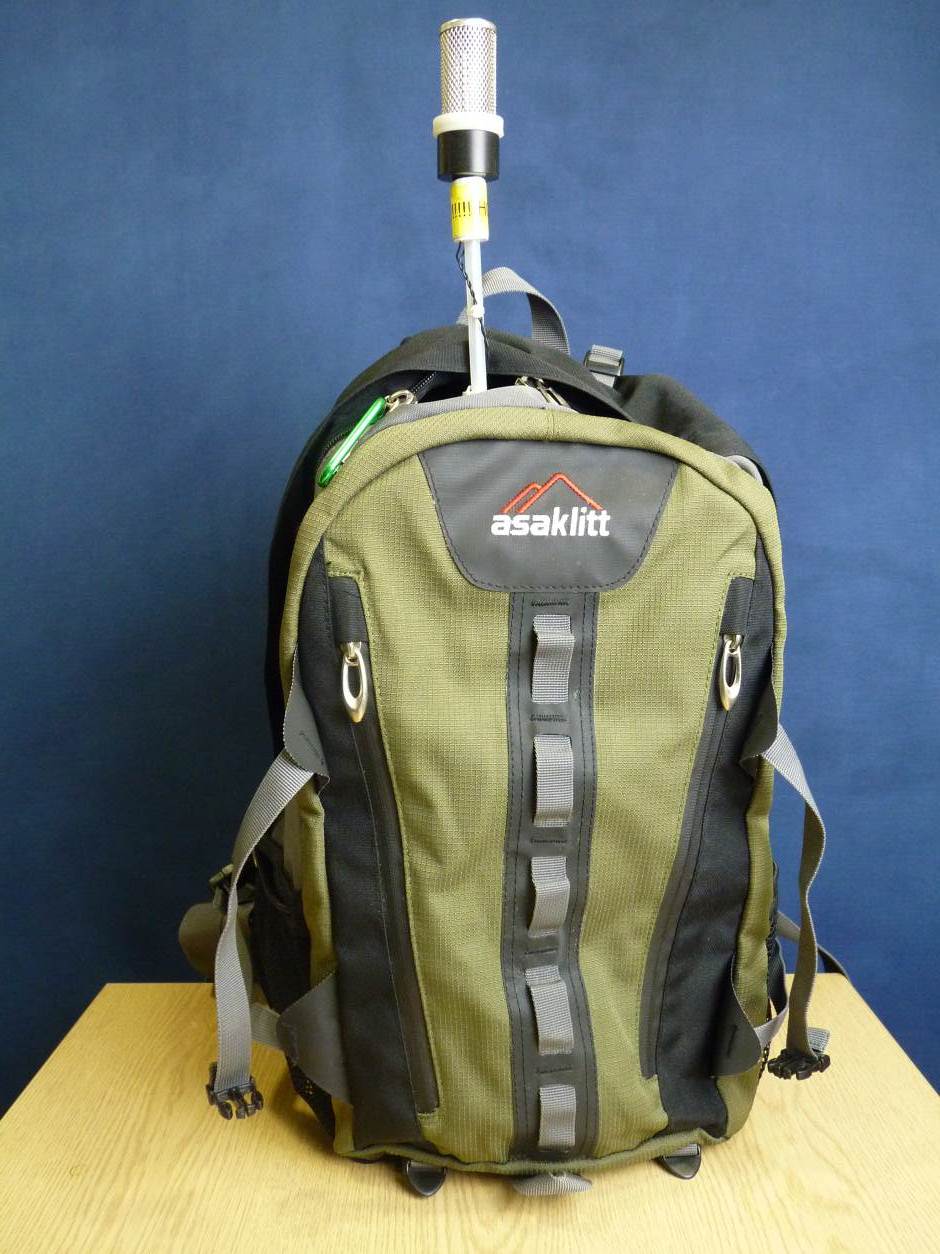


Figure S4: Housing located in 30 L back-pack with heated inlet protruding.


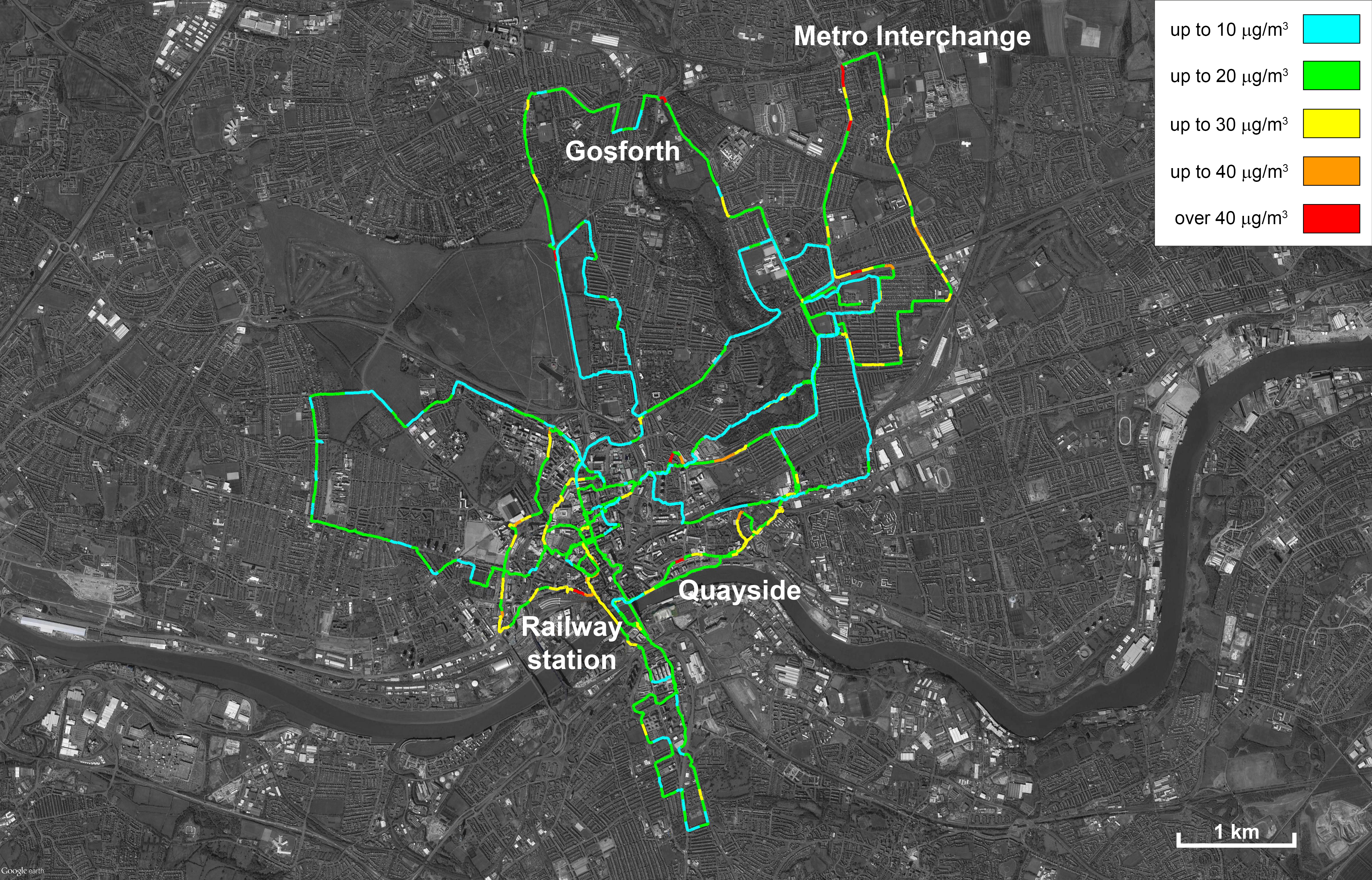
Figure S5: Annually normalised PM_10_ concentrations across Newcastle upon Tyne and Gateshead, UK, collected using a DustMate monitor with heated inlet during the period 09/06/15 to 26/06/15. Map produced using Google Fusion Tables and Google Earth Pro. Map data: Google, Landsat.


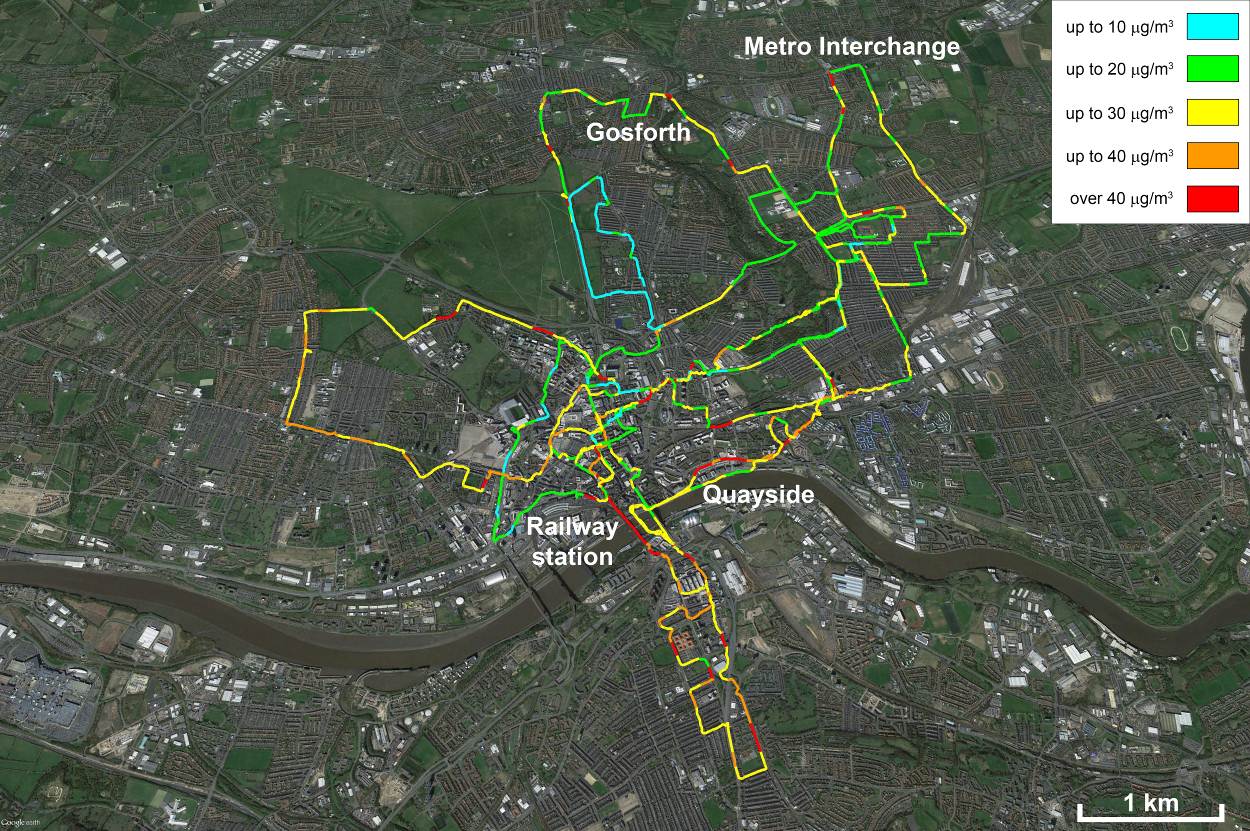
Figure S6: Raw data for PM_10_ concentrations across Newcastle upon Tyne and Gateshead, UK, collected using a DustMate monitor with heated inlet during the period 16/06/15 to 09/06/15. Map produced using Google Fusion Tables and Google Earth Pro. Map data: Google, Landsat.


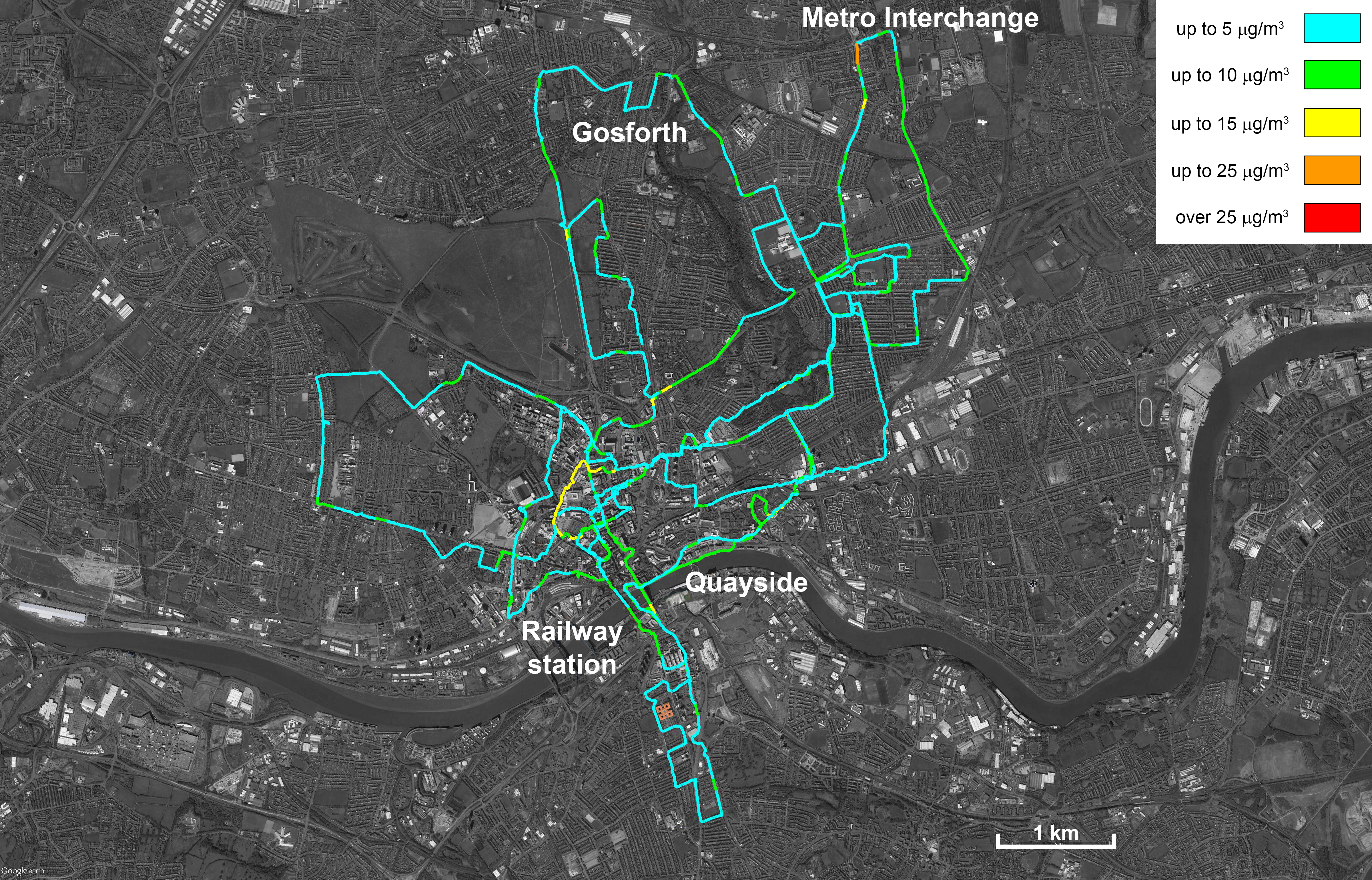
Figure S7: Annually normalised PM_2.5_ concentrations across Newcastle upon Tyne and Gateshead, UK, collected using a DustMate monitor with heated inlet during the period 09/06/15 to 26/06/15. Map produced using Google Fusion Tables and Google Earth Pro. Map data: Google, Landsat.


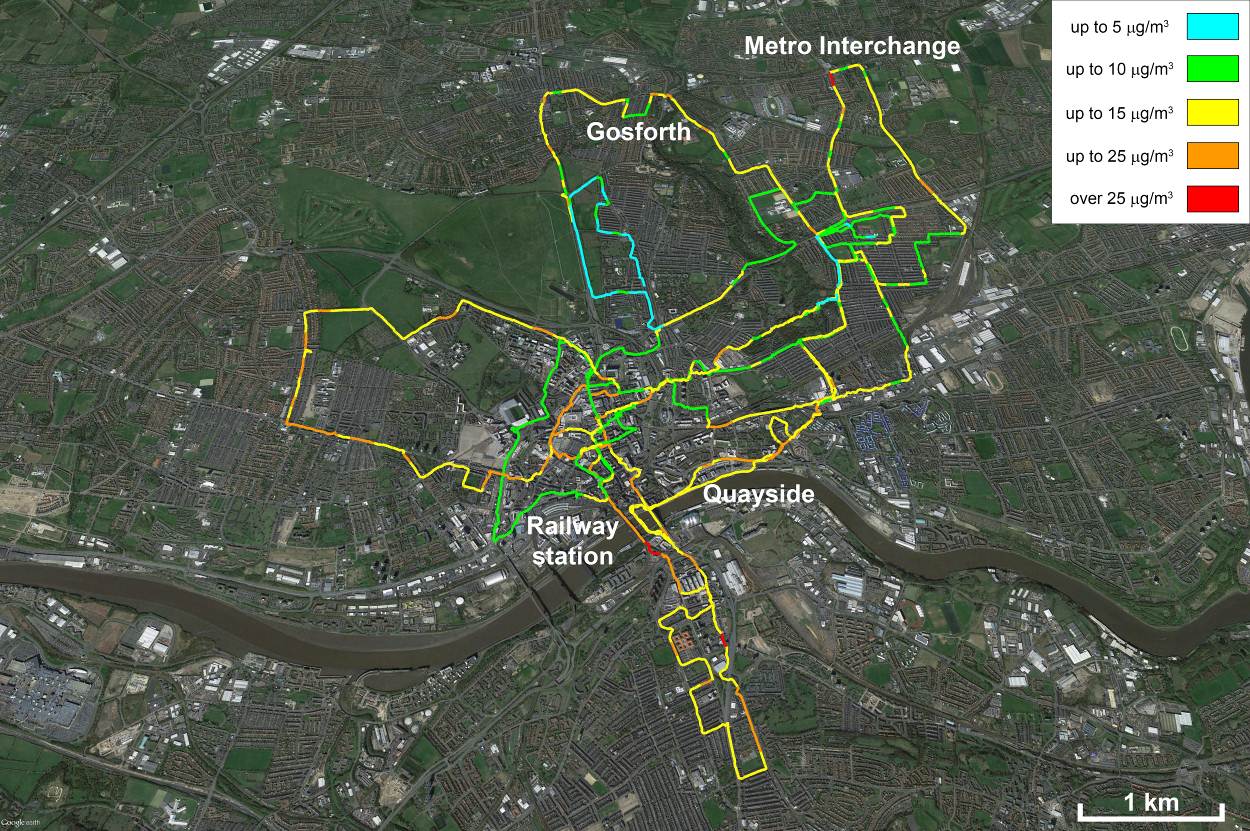
Figure S8: Raw data for PM_2.5_ concentrations across Newcastle upon Tyne and Gateshead, UK, collected using a DustMate monitor with heated inlet during the period 16/06/15 to 09/06/15. Map produced using Google Fusion Tables and Google Earth Pro. Map data: Google, Landsat.
